# Therapeutic potency of curcumin on radiodermatitis: A systematic review

**DOI:** 10.22038/AJP.2023.23175

**Published:** 2024

**Authors:** Hossein Abdeahad, Nikoo Saeedi, Afsane Bahrami, Abdulridha Mohammed Al-Asady, Saeide Mansoori, Amir Avan, Majid Khazaei, Elnaz Ghorbani, Mikhail Ryzhikov, Seyed Mahdi Hassanian

**Affiliations:** 1 Department of Clinical Biochemistry, Faculty of Medicine, Mashhad University of Medical Sciences, Mashhad, Iran; 2 Student Research Committee, Islamic Azad University, Mashhad Branch, Mashhad, Iran; 3 Clinical Research Development Unit, Faculty of Medicine, Imam Reza Hospital, Mashhad University of Medical Sciences, Mashhad, Iran; 4 Department of Medical Sciences, Faculty of Nursing, Warith Al-Anbiyaa University, Iraq; 5 Department of pharmacology, Faculty of Medicine, Mashhad University of Medical Sciences, Mashhad, Iran; 6 Department of Medical Sciences, Faculty of Dentistry, University of Kerbala, Iraq; 7 Metabolic Syndrome Research Center, Mashhad University of Medical Sciences, Mashhad, Iran; 8 Department of Human Genetics, Faculty of Medicine, Mashhad University of Medical Sciences, Mashhad, Iran; 9 Department of Medical Physiology, Faculty of Medicine, Mashhad University of Medical Sciences, Mashhad, Iran; 10 Department of Medical Microbiology and Virology, Faculty of Medicine, Mashhad University of Medical Sciences, Mashhad, Iran; 11 Saint Louis University, School of Medicine, St. Louis, MO, USA; 12 Applied Biomedical Research Center, Mashhad University of Medical Sciences, Mashhad, Iran; † Equal first author

**Keywords:** Radiotherapy, Dermatitis, Turmeric, Curcuma, Cancer

## Abstract

**Objective:**

Radiodermatitis (RD) is a frequent adverse event of radiotherapy (RT). Currently, there is no consensus and approved protocol for the treatment of RD. Curcumin (CUR) is a natural polyphenol obtained from turmeric and it has low intrinsic toxicity in humans. The aim of this systematic review was to explore the efficacy of CUR for prevention and treatment of RD.

**Materials and Methods:**

A systematic literature review was performed in the following online databases: Cochrane library, PubMed, Scopus, Web of Science, MEDLINE, and EMBASE. Among the 5 selected records, 3 had a randomized clinical trial (RCT)-design and the other had a pilot and controlled study designed. The included studies were performed on breast cancer (N=3), head and neck cancers (N=1) and different types of cancer (N=1).

**Results:**

Four of the studies reported that the application of curcumin in cancer patients undergoing radiotherapy is associated with decreased intensity of radiodermatitis. However, one study did not report any significant effect of CUR on radiodermatitis. This review provides substantial evidence which confirm the clinical value of CUR in cancer supportive care.

**Conclusion:**

Further prospective clinical trials in larger scales are warranted in order to determine the " supplemental form and dose of CUR" for RD prevention and treatment in patients receiving radiotherapy.

## Introduction

Radiodermatitis (RD) is a frequent adverse event of radiotherapy (RT) (Ryan et al., 2013). RD results from damage to DNA, and modifications in the structure of proteins, lipids, or carbohydrates. Accumulation of these changes leads to injury and destruction of epidermal basal cells (Zhang et al., 2013). The clinical presentations of RD include a wide range including erythema, xeroderma, hyperhidrosis, dyspigmentation, telangiectasias, hair loss, deep ulcers, fibrosis and necrosis (McQuestion, 2006). Acute RD influences the quality of life. In patients with severe forms of RD, unplanned gaps may occur during treatment and interfere with treatment plan (Bataini et al., 1988).

Management of severe forms of RD is very vital in cancer patients requiring curative radiotherapy. Currently, there is no consensus and approved protocol for the treatment of RD except using lukewarm water and lenient soap (Campbell and Illingworth, 1992; Roy et al., 2001; Lavery, 1995). Recently, moisturizing creams, anti-inflammatory agents, silymarin, *Aloe vera* gel, marigold, and curcumin (CUR) have been investigated for their therapeutic potencies. Results of previous studies suggest these agents as palliative treatment of RD (Falkowski et al., 2011; Heggie et al., 2002; Pommier et al., 2004). 

Considerable efforts have been performed to investigate the efficiency of topical compounds in the treatment of RD (Tables 1 and 2). Results of a meta-analysis study that performed in 2013 demonstrated the therapeutic and prophylactic efficacy of several agents such as corticosteroids trolamine, gentian violet, sucralfate, *Aloe vera*, biafine, urea, mixture of oil and aqueous, vitamin C, and hyaluronic acid on treatment of radiodermatitis (Zhang et al., 2013).

CUR (1,7-bis(4-hydroxy-3-methoxyphenyl)1,6-heptadiene-3,5-dione) is a natural polyphenol obtained from turmeric (*Curcuma longa *L.), with low intrinsic toxicity in humans (Hosseini et al., 2017). CUR is known for its anti-microbial, anti-cancer, anti-inflammatory, and chemotherapeutic properties (Tajbakhsh et al., 2017; Sahebkar, 2014; Shafiee et al., 2017; Najafi et al., 2015; Arshami et al., 2013; Amini et al., 2023). Interestingly, CUR is able to suppress enzymes mediating the production of reactive oxygen species (ROS), inflammatory lipids, pro-inflammatory transcription factors, at both protein and gene levels and upregulates the expression level of anti-oxidant enzymes (Ryan et al., 2013).

CUR inhibits amyloid fibril formation. Due to this property, CUR is utilized for the treatment of common skin diseases including eczema, acne, and skin crease. Also, a number of experimental studies have approved its prophylactic role in UV-induced skin tumorigenesis (Dwivedi and Abu-Ghazaleh, 1997; Dwivedi et al., 2003; Dwivedi et al., 2005). However, the therapeutic properties of CUR in humans is still inconsistent and without a general agreement (Palatty et al., 2014; Wolf et al., 2017). 

This study aims to explore the efficacy of CUR for prevention and treatment of RD by searching for evidence through a systematic review. Considering the multi-functional and strategic role of CUR in reduction of inflammation and oxidation, utilizing this agent in RD treatment may improve clinical management and patient-related outcomes. 

## Materials and Methods

**Search strategy and study selection**

We employed multiple databases to find literature on the effect of purified CUR or curcumin-containing mixtures or standardized *Curcuma spp*. extracts on RD. Human interventional studies which investigated radiation dermatitis severity or intensity in both intervention and comparator groups at basal level and the end of intervention, were eligible for inclusion. 

**Figure 1:**
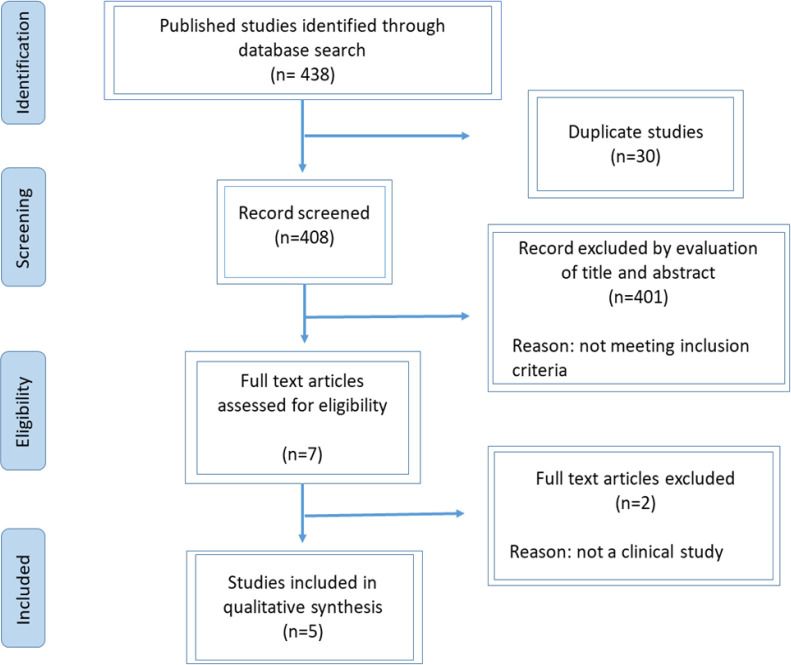
Flowchart of literature search and selection process

The systematic literature review restricted to English language was performed in the following online data bases: Cochrane library, PubMed, Scopus, Web of Science, MEDLINE, and EMBASE. The following keywords were applied for the search: ‘curcumin’, ‘*curcuma*’, ‘turmeric’, ‘*curcuma domestica*’, ‘*Curcuma longa *L’, ‘radiotherapy’, ‘dermatitis’, ‘radiodermatitis’ and ‘radiation dermatitis’. Figure 1 shows the summary of systematic search with details. Also, a manual search through the reference lists of the included articles and relevant papers was performed. The articles with irrelevant titles, review papers, conference abstracts, case reports, and experimental studies were excluded. Screening and selection of articles to be entered in the systematic review were independently performed by 2 expert reviewers. Discrepancies in the included papers were resolved by discussion with supervisors.

**Data extraction**

Our favorable outcomes were Radiation Dermatitis Severity (RDS), signs of skin damage, and the incidence of side effects. The following information was retrieved from the included studies: year of publication, country, type of study, type of cancer, mean dose of radiation, number of patients, age, dose and duration of treatment with CUR. Furthermore, mean±SD of RD manifestation degree (RDS score or incidence and number of side effects) at basal time and at the end of intervention were gathered.

## Results

**Literature review**

A total of 438 papers were recognized. About 91% of these papers were omitted after deleting duplicates and screening of the titles and abstracts. After reviewing the full text of the remaining articles, five articles were obtained for analysis (Figure 1).

Among the 5 selected records, 3 had a randomized clinical trial (RCT)-design (Ryan et al., 2013; Rao et al., 2017; Wolf et al., 2017) and two others had a pilot and controlled study design (Palatty et al., 2014; Belcaro et al., 2014). Three studies were conducted among breast cancer patients (Ryan et al., 2013; Rao et al., 2017; Wolf et al., 2017), one study examined head and neck cancer patients (Palatty et al., 2014), and the other one was conducted on different cancers (Belcaro et al., 2014). 

Duration of treatment among the studies ranged between 5 to 7 weeks. There was a wide spectrum of radiation doses (30-66 Gy). Only one study used purified CUR for supplementation (Ryan et al., 2013). Two studies utilized curcuminoids (containing CUR, demethoxy CUR, and bisdemethoxy CUR) (Belcaro et al., 2014; Wolf et al., 2017) and the remaining two studies utilized Vicco turmeric cream (VTC), containing turmeric and sandal wood oil (*Santalum album *L) (Palatty et al., 2014; Rao et al., 2017). Three articles administrated placebo (Ryan et al., 2013; Belcaro et al., 2014; Wolf et al., 2017). In two studies moisturizing cream or Johnsons Baby Oil (JBO) (Palatty et al., 2014; Rao et al., 2017) were prescribed as the comparator group.

Results of Rao and colleagues showed that the usage of CUR-based cream among breast cancer patients led to delay and mitigation in radiodermatitis (Rao et al., 2017). In line with this, Palatty et al. (Palatty et al., 2014) and Ryan et al. (Ryan et al., 2013) found that CUR reduced the intensity of RD. In contrast with this, Wolf and colleagues showed that CUR could not reduce RD severity (Ryan Wolf et al., 2018). Belcaro and colleagues reported that CUR could successfully reduced the radiotherapy-related side effects in different types of cancer (Belcaro et al., 2014). 

## Discussion

Finding from the current systematic review could provide evidence of the beneficial effect of CUR on the improvement of RD in cancer patients receiving RT. Consistently, preclinical and experimental studies have shown that CUR supplementation therapy was associated with improved outcomes in the treatment of ulcer, dermatitis and papilloma formation in mice with radiation exposure. The pathogenesis of RD includes production of ROS and damage to DNA (Stone et al., 2003; Schaue et al., 2012). CUR increases the expression of enzymes like catalase, superoxide dismutase, glutathione transferase, and glutathione-peroxidase, at both protein and mRNAs levels. A great body of evidence has indicated that CUR can scavenge reactive oxygen and nitrogen species (Baliga et al., 2013; Gupta et al., 2013; Najafi et al., 2015). Also, results obtained from *in vivo* and *in vitro* studies supported CUR’s antioxidant functions and its critical role in prevention of lipid peroxidation and DNA degradation (Jelveh et al., 2013; Parshad et al., 1998; Ghasemi et al., 2022). CUR enhanced the repair and regeneration of wounds and re-epithelialization of the epidermis, decreased mean healing duration, increased neovascularization, and upregulated production and deposition of collagen at the injury site (López-Jornet et al., 2011; Jagetia and Rajanikant, 2004; Jagetia and Rajanikant, 2005). 

Moreover, CUR has shown significant anti-inflammatory effects in cutaneous tissues (Huang et al., 1997). According to the literature, CUR inhibits ornithine decarboxylase responses, DNA synthesis, epidermal lipoxygenase and cyclooxygenase (COX) activity, and activation of inflammatory pathways including extracellular signal-related kinase (ERK) and nuclear factor kB (NF-kB) signaling pathways in stimulated cells (Chun et al., 2003; Baliga et al., 2013; Thangapazham et al., 2013; Gupta et al., 2013; Ghasemi et al., 2022; Shojaei et al., 2023). Furthermore, CUR significantly downregulates both acute and chronic skin inflammatory reactions and induces protective effects by decreasing early-releasing cytokines and interleukins including tissue necrosis factor- α (TNF-α), lymphotoxin-β, transforming growth factor beta (TGF-β), hypoxia-inducible growth factor-1α, transcription factor Egr-1(Egr-1), stromal cell-derived growth factor-1α, and hemeoxygenase-1 in epidermis (Okunieff et al., 2006). Previous studies demonstrated that CUR suppresses the induction of immediate early response genes in endothelial cells fibroblasts (Pendurthi and Rao, 2000; Chen et al., 2006).

The current systematic review has several limitations. There were few eligible records, and most had small sample sizes (<40 subjects). In spite of small number of patients in the included studies, the ongoing trials (Table 1), have recruited large number of patients which may produce more reliable results. 

**Table 1:** A review of clinical trials investigating therapeutic role of curcumin in radiodermatitis

**Study (Year)**	**Registration number**	**Phase**	**Subjects enrolled**	**Type of cancer**	**Location**	**Follow up period**	**Status**
Morrow (2015)	NCT02556632	II	191	Non-inflammatory breast cancer	University of Rochester	1 week	Completed
Ryan (2010)	NCT01246973	II	686	Breast cancer	University of Rochester	6 weeks	Completed
Heydari (2019)	IRCT20181208041882N3	III	52	Breast cancer	Yazd University of Medical Sciences	4 weeks	Ongoing

**Table 2:** Characteristics of included studies

**Author, year**		Ryan 2013 (Ryan et al. 2013)	Palatty 2018 (Palatty et al. 2014)	Stone 2003 (Belcaro et al. 2014)	Ryan wolf 2018 (Rao et al. 2017)	Wolf 2017 (Wolf et al. 2017)
**Country**		USA	India	Italy	India	USA
**Design**		Double Blind RCT	Pilot	Controlled study	Investigator-blinded RCT	Double-blinded RCT
**Duration of trial**		7 weeks	7 weeks	60 days	5 weeks	During course of RT until one-week post RT
**Type of cancer**		Breast	Head/Neck	All types	Breast	Breast
**Intervention**	**Case**	Curcumin (2.0 grams, 3 times daily)	VTC (2 g, 5 times daily)	Curcuminoids (100 mg, 3 times daily)	VTC (5 gr, 5 times daily)	Curcuminoids (500 mg, 3 times daily)
	**Control**	Placebo	Moisturizing cream, JBO (2 ml, daily)	Placebo	Moisturizing cream, JBO (5ml, 5 times daily)	Placebo
**Sample size**	**case**	14	22	40	20	283
	**control**	16	24	40	20	295
**Age (year)**		58.1±2.2	56.9±7.21	55.8±3.3	50.93±9.52	57.6±0.4
**Race**		White/Caucasian, Black/African American, Multiracial	NR	NR	NR	White/Caucasian, Black/African American, Multiracial
**Administration route**		Oral	Topical	Oral	Topical	Oral
**Mean radiation dose (Gy)**		46.51±3.48	66.0±5.70	30-50	50	48.34±0.14
**Assessed measurements**		RDS score	Signs of skin damage	The incidence of side effects	Dermatological analysis based on the criteria of (RTOG/EORTC)	RDS score


Abbreviations: RT (radiation therapy), JBO (Johnsons Baby Oil), VTC (Vicco Turmeric Cream), RDS (Radiation Dermatitis Severity), RTOG/EORTC (Radiation Therapy Oncology Group/ European Organization for Research and Treatment Cancer), NR (Not reported).

Furthermore, there were significant variations among the included studies in terms of study type, demographic characteristics, tumor types, mean radiation dose, supplement form, dose, and duration. However, evaluation of the therapeutic potential of CUR in healing dermatitis following radiation therapy in patients with breast cancer is an issue that has received much attention. The majority of the published data and ongoing trials are focused on this topic. Regarding the considerable number of breast cancer patients, the effect of CUR on RD in these patients may present valuable findings. 

The included clinical trials did not mention CUR dose, which can be considered as an important limitation of this study can be since the “effective dose” of CUR for severe RD is not identifiable. However, the 6.0 g daily dose of CUR certainly reduced the rate of adverse reactions and detection of circulating CUR. Although 6.0 g of CUR is an accepted and routine dose, it is plausible that a megadose of CUR (up to 8 grams daily), may act more efficiently against RD severity. None of the selected studies administrated bioavailability-improved formulations of CUR, except one study in which CUR was co-administered with lecithin as an absorption enhancer (Belcaro et al., 2014). One major problem of CUR, is its miserably low oral bioavailability, particularly from non-dietary pharmaceutical complexes, with the need to increase its concentration in patients. Therefore, the development of better formulations of CUR could have an exciting effect on compliance, making it easy to systematically evaluate the clinical importance of this compound in cancer best supportive care.

In conclusion, our systematic review of the evidence of eligible studies presented that CUR supplementation has significant beneficial effects on RD severity. This review provides substantial evidence confirming the clinical value of CUR in cancer supportive care. Further prospective clinical trials in larger scales are warranted in order to determine the "real effective extract, supplemental form and dose of CUR" for RD prevention and treatment in patients receiving radiotherapy.
